# Supplementary material for: Inflammatory factors and risk of lung adenocarcinoma: a Mendelian randomization study mediated by blood metabolites
Source: Front Endocrinol (Lausanne). 2024 Aug 27;15:1446863. doi: 10.3389/fendo.2024.1446863 (PMC11384989; doi:10.3389/fendo.2024.1446863)
Supplement: Supplementary file 1 [file DataSheet1.zip › supplementary files/S2/GCST90200324/sensitivity-analysis.pdf]

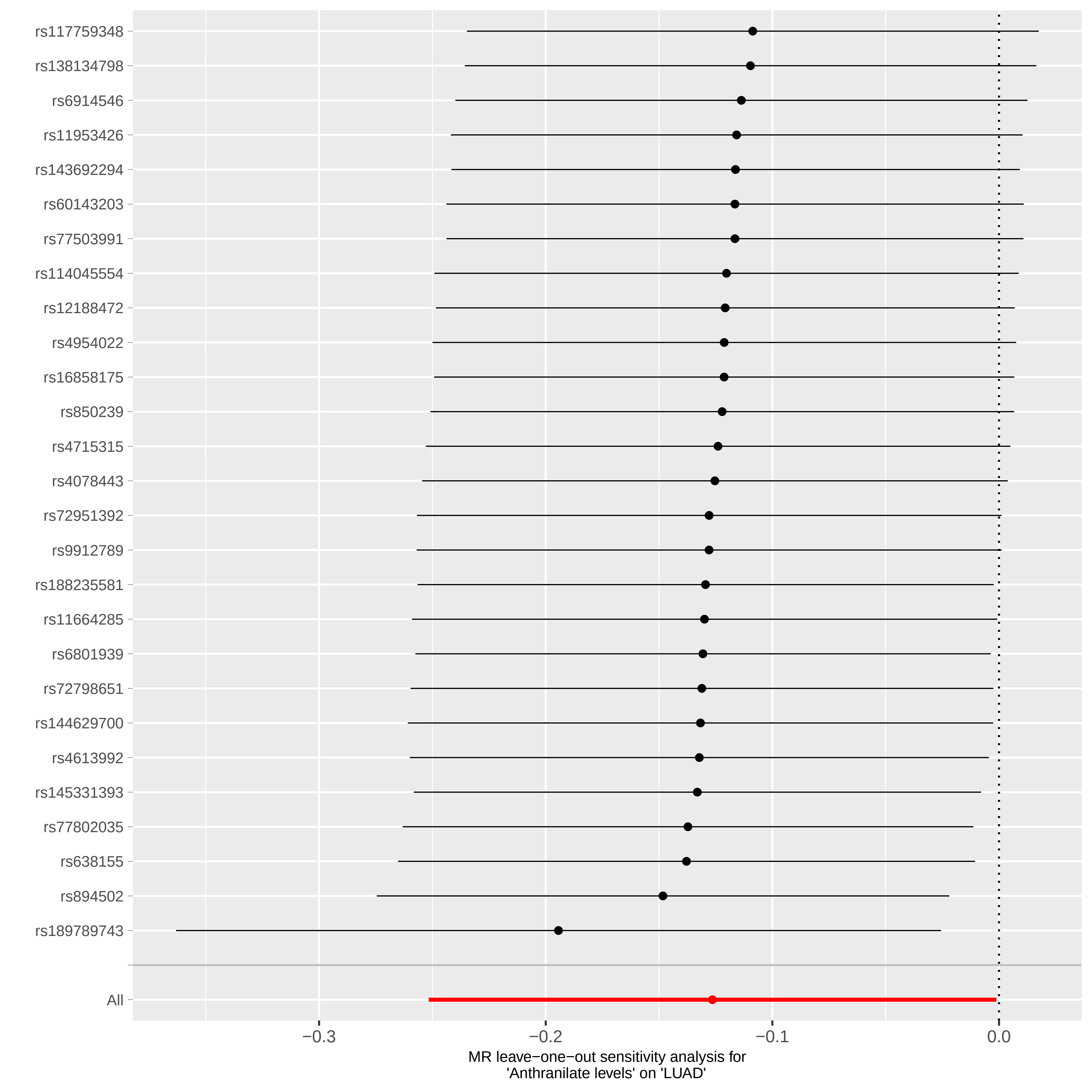
rs117759348
rs138134798
rs6914546
rs11953426
rs143692294
rs60143203
rs77503991
rs114045554
rs12188472
rs4954022
rs16858175
rs850239
rs4715315
rs4078443
rs72951392
rs9912789
rs188235581
rs11664285
rs6801939
rs72798651
rs144629700
rs4613992
rs145331393
rs77802035
rs638155
rs894502
rs189789743
All
−0.3
−0.2
−0.1
0.0
MR leave−one−out sensitivity analysis for
'Anthranilate levels' on 'LUAD'
